# Anemia prevalence, severity, types, and correlates among adult women and men in a multiethnic Iranian population: the Khuzestan Comprehensive Health Study (KCHS)

**DOI:** 10.1186/s12889-022-12512-6

**Published:** 2022-01-25

**Authors:** Elham Akbarpour, Yousef Paridar, Zahra Mohammadi, Ali Mard, Leila Danehchin, Farhad Abolnezhadian, Shima Azadpour, Zahra Rahimi, Mohammad Zamani, Bahman Cheraghian, Hossein Poustchi, Ali-Akbar Shayesteh

**Affiliations:** 1 Liver and Pancreatobiliary Diseases Research Center, Digestive Disease Research Institute, Tehran University of Medical Sciences, Shariati Hospital, N. Kargar St., 14117 Tehran, Iran; 2 School of Medicine, Dezful University of Medical Sciences, Dezful, Iran; 3 Alimentary Tract Research Center, Imam Khomeini Hospital Clinical Research Development Unit, School of Medicine, Ahvaz Jundishapur University of Medical Sciences, Ahvaz, Iran; 4 Behbahan Faculty of Medical Sciences, Behbahan, Iran; 5 Shoshtar Faculty of Medical Sciences, Shoshtar, Iran; 6 Abadan Faculty of Medical Sciences, Abadan, Iran; 7 Department of Epidemiology, School of Public Health, Iran University of Medical Sciences, Tehran, Iran; 8 Digestive Diseases Research Institute, Tehran University of Medical Sciences, Tehran, Iran; 9 Alimentary Tract Research Center, Clinical Sciences Research Institute, Department of Biostatistics and Epidemiology, School of Public Health, Ahvaz Jundishapur University of Medical Sciences, Ahvaz, Iran; 10 Alimentary Tract Research Center, Imam Khomeini Hospital Clinical Research Development Unit, Department of Biostatistics and Epidemiology, School of Public Health, Ahvaz Jundishapur University of Medical Sciences, Ahvaz, Iran; 11 Gastroenterology Department, Imam Khomeini Hospital, Azadegan Ave, Ahvaz, Iran

**Keywords:** Anemia, Anemia severity, Anemia types, Population-based study, Public health, Epidemiology, Prevalence, Associated factors, Iran

## Abstract

**Background:**

Despite all recent health-related improvements, anemia remains an extensive global public health issue affecting the lives of about one-fourth of the world population in a geographically heterogeneous pattern. We, therefore, aimed to illustrate the prevalence, severity, most common types, and major determinants of anemia among adults in Khuzestan, Iran, from 2016 to 2019.

**Methods:**

In a large population-based cross-sectional study comprising of a diverse population, each participant underwent a questionnaire-based interview and laboratory testing for hematological analysis. A hemoglobin (HGB) concentration of < 12 g/dL in non-pregnant women and < 13 g/dL in men were defined anemic. The multivariate logistic regression analysis was performed to explore the association between anemia and its potential determinants.

**Results:**

Data on 29,550 (96.87%) males and non-pregnant females between 20–65 years of age (mean age: 41.90 ± 11.88 years; female sex: 63.58%; Arab ethnicity: 48.65%), whose HGB level was available, were included in the study. The mean ± SD HGB concentration was 13.75 ± 1.65 g/dL. The age- and sex-standardized prevalence rate of anemia was 10.86% (95% CI: 10.51–11.23%). The most prevalent degree was mild anemia (7.71%, 95% CI: 7.40–8.03%) and only 0.17% were severely anemic. Of those considered anemic, the highest proportion was related to normochromic/microcytic (50.65%), followed by hypochromic/microcytic (30.29%).

In the multiple logistic regression, the parameters of female gender (OR: 3.17, 95% CI: 2.68–3.76), age group of 35–49 years (OR: 1.66, 95% CI: 1.52–1.82), being underweight (OR: 1.58, 95% CI: 1.29–1.93), being unemployed or retired (OR: 1.55, 95% CI: 1.33–1.81), and living in urban areas (OR: 1.18, 95% CI: 1.09–1.29) were major determinants of anemia. Additionally, we observed a minor but significant positive association between anemia status and CKD, older ages, increased night sleep duration, being a housewife and married, as well as a negative association between anemia and factors including hookah smoking, presence of metabolic syndrome, and overweight and obesity.

**Conclusions:**

Taken together, the anemia prevalence in this study population was of mild public health significance. The major suspected causes might be iron deficiency and chronic disease anemias. Comparably higher rates of anemia were observed amongst women, individuals aged 35–49 years, underweights, unemployed or retired subjects, and urban residents.

**Supplementary Information:**

The online version contains supplementary material available at 10.1186/s12889-022-12512-6.

## Background

As defined by the World Health Organization (WHO), anemia is a condition in which the red blood cells (RBCs) count or the within hemoglobin (HGB) content falls below an established reference range (< 12 g/dL in non-pregnant women and < 13 g/dL in men), resulting in an impaired oxygen-carrying capacity to meet the tissues’ physiological demands [1]. However, the optimal threshold for healthy individuals may vary due to personal and environmental conditions.

Anemia is regarded as the most prevailing micronutrient deficiency disorder worldwide, mainly in the poor socioeconomic strata of societies and as a result of poverty [2, 3]. According to the estimates derived from the Global Burden of Diseases, Injuries and Risk Factors 2013 Study (GBD 2013), anemia affects the lives of nearly 1.93 billion people worldwide, corresponding to 27% of the global population [4]. The prevalence rate of anemia especially maximizes in low- and middle- income countries, and mostly among under-5 children, pregnant and lactating women, and the elderly [3]. Although, there exists a slight downward trend from 1990 to 2010 in absolute prevalence and years lived with disability (YLD) caused by anemia, a great physical burden (61.5 million YLDs) and health-care cost (additional $7,000-$30,000 than the same condition without anemia) is still inflicted on the health systems [3, 5]. Iran is an upper-middle-income country in the middle east, with an estimated age-standardized prevalence rate of 23% based on GBD 2013, [4] which is classified as moderate by WHO regarding the anemia prevalence [6]. The estimated anemia frequency in Iran varies between 10–30%, specifically higher in children and adolescents, females, and older ages [7].

An intricate interplay of several concomitant factors (such as biological factors, demographics, lifestyle, personal habits, socioeconomic status, and geographical differences) may affect the distribution and severity, the pattern of the underlying pathophysiology, vulnerability to, and consequences of anemia [8, 9]. Iron deficiency is the most common etiology of anemia and responsible for more than half of anemia cases worldwide [3]. The other possible contributors of anemia in low- and middle-income countries include the shortage of non-iron hematopoietic nutrients (folic acid, vitamin B12, and vitamin A), parasitic diseases and infections (malaria, hookworm infections, and schistosomiasis), inherited hemoglobinopathies (sickle cell disease and thalassemia), chronic diseases (cancer, autoimmune disease, chronic kidney disease, and congestive heart failure) [8].

Anemia can be troublesome both as being a sign of a serious underlying health condition, such as cancer, or by causing symptoms itself. Untreated anemia could cause low energy, strength, and productivity, presenting as chronic fatigue, lethargy, concentration difficulty, low blood pressure, shortness of breath, and even impaired mental well-being [10–13]. Despite all recent social and economic development and health-related improvements, anemia remains an extensive global public health issue affecting individuals at any stage of life from both developing and developed countries and causing serious impacts on quality of life, morbidity, and mortality [2].

Given the burden inflicted on the health system by anemia, it is crucial to modify the health-care policies in a way that enables some appropriate intervention measures against anemia. To our knowledge, of a sparse number of studies attempting to estimate the prevalence of anemia in Iran, most are restricted to children, adolescents, and women of childbearing age, whether pregnant or not, and such studies lack consistency and community representation [7, 14–16]. As stated in these studies, anemia has heterogeneous patterns geographically and across different populations in terms of epidemiological indicators, [7] so it is important to precisely identify their prevalence and region-specific contributors both at the national and regional levels to be able to implement a more relevant and efficient plan. Population-based cross-sectional studies serve as plausible evidence in this regard. Correspondingly, the Khuzestan Comprehensive Health Study (KCHS) was established between 2016 and 2019 to create the framework needed for priority setting in health policymaking and research [17]. As a part of the KCHS, we aimed to illustrate the overall and age- and sex-standardized prevalence, severity, most common types, and major associated risk factors of anemia among adult men and non-pregnant women at the provincial level in Khuzestan, Iran, from 2016 to 2019. This report is one of the most comprehensive extensive population-based studies of anemia in Iran.

## Methods

### Design and setting

In a descriptive analytics cross-sectional study, as a part of the KCHS study, we reported the overall and age- and sex-standardized prevalence, severity, most common types, and determinants of anemia among adult men and non-pregnant women at the provincial and county-level. The KCHS is a large population-based cross-sectional study comprising of about 30,000 Iranian adults from 27 counties of Khuzestan province, southwest of Iran, which aims to assess several health aspects, including the burden of NCDs, as well as identifying their contributors. This project was funded by the National Institute for Medical Research Development (NIMAD) (grant number: 940406) in partnership with Iranian Blood Transfer Organization (IBTO). It was first launched in October 2016 with the cooperation of Digestive Diseases Research Institute (DDRI), Jundishapur and Dezful universities of medical sciences, and Abadan and Behbahan faculties of medicine. The enrollment lasted up to November 2018. Details of the methods on data collection, protocols, and study design have already been presented elsewhere [17]. The investigators of the DDRI affiliated to Tehran University of Medical Sciences (TUMS), Tehran, Iran, prepared this report in October 2020. The KCHS protocol was approved by the ethics committees of the NIMAD (IR.NIMAD.REC.1394.002), and was conducted in accordance with the Declaration of Helsinki. All participants have authorized using the recorded data for scientific goals.

### Study population, and sample size

Khuzestan is a southwestern province of Iran, bordering Iraq and the Persian Gulf, with near 5 million estimated inhabitants of various ethnicities, cultures, lifestyles, and socioeconomic groups from 27 counties. The climate is generally hot semi-arid or desert, and it is covered by two different landforms, mountainous in the north and flat in the south. This province of Iran has been geopolitically exposing to numerous health threats, such as war, air pollution, oil resources, dust phenomena, and extreme high temperatures. The KCHS was based on about 0.6% of this population from all 27 distinct counties of Khuzestan. Overall, 30,506 male and female adults between 20–65 years of age from both urban and rural regions were included to the KCHS. No exclusion criteria, other than an unwillingness to participate in the study or having mental or physical disabilities confounding the completion of questioning process, were used. In a stratified random sampling approach, a number of Health Houses (primary care centers) in each of the 27 counties were selected based on the proportion of the total province population living in that county. Thereafter, 30 random individuals who fulfilled the eligibility criteria were recruited from the population census at each Health House. The estimated sample size is large enough to specify the prevalence of anemia in this region.

### Data collection, variables, and measurements

The data collection process of the KCHS is summarized as follows: Based on a stratified random multistage sampling plan, the target participants were selected and visited face-to-face by a trained staff member. After providing an adequate explanation about the aim of research to the subjects, they were invited to the study sites if they agreed to participate. On the morning of the visit day, a written informed consent was obtained initially. Firstly, venous blood samples were taken from each subject after 8 to 12 h of fasting, and then, anthropometric indices and blood pressure were measured. The samples were refrigerated in cool boxes, shipped to the reference laboratory within three hours, and analyzed for hematological and biochemical parameters. Finally, each individual was interviewed and a standardized questionnaire regarding general, medical condition, was completed for all participants (Additional file 2). Additionally, nutritional information, as well as, few other specific conditions (such as gastrointestinal and psychological variables), were recorded for a subsample. A quality assurance and quality control team was assembled to regularly review the data collection procedures.

All required data for this report were extracted from the database of the KCHS and are outlined in Additional file 1 by the definitions and categories of each variable.

The anemia status applied in our report was accorded with the WHO definition of anemia, [1] i.e. a HGB concentration of < 12 g/dL in non-pregnant women and < 13 g/dL in men. In addition, the anemia severity was categorized into three groups of mild (HGB: 11–11.9 g/dL in women and 11–12.9 g/dL in men), moderate (HGB: 8–10.9 g/dL), and severe anemia (HGB < 8 g/dL). The anemia type was classified by the following hematimetric indices (Additional file 1): Mean Corpuscular Hemoglobin Concentration (MCHC); Mean Corpuscular Volume (MCV) [11].

In this study, the primary outcome was to assess the crude and age- and sex-standardized prevalence rate of anemia among adult men and non-pregnant women population of Khuzestan. Secondly, the respective figures were specifically explored to determine the potential associated factors. Finally, we studied the characteristics of anemia, like the distribution of anemia severity and major clinical types of anemia, in age-specific subgroups by gender.

### Statistical analysis

The database was first structured using Microsoft Office Excel 2016 and all statistical analyses were conducted using Stata/SE version 12 (StataCorp, Texas, USA). The graphs were created using Microsoft Office Excel 2016. Additionally, ArcGIS version 10.8 was used to generate the county-level age- and sex-standardized prevalence rates in the study area map. Frequency, percentage, and 95% Confidence Interval (95% CI) were used as descriptive statistics for categorical variables to summarize the characteristics of the anemic study population. Moreover, we estimated age- and sex-standardized prevalence rates of anemia using the age and sex structure of the Khuzestan population census the year 2015. We visualized the distribution of the anemia status among men and women by age. Anemia prevalence (crude and age- and sex-standardized) rates were reported as proportions along with 95% CI for various subgroups. For comparison between these groups of explanatory variables by anemia levels, the chi-squared test was performed to explore unadjusted associations. The severity and different types of anemia were compared between gender and age groups, as well. The crude association between anemia and its potential determinants was assessed using the univariate logistic regression analysis. Explanatory variables that were found to have a significant association with anemia in the univariate analysis, were further adjusted through multivariable logistic regression models using a forward stepwise approach. The Odds Ratios (ORs) and their 95% CI were calculated by logistic regression analysis to measure the strength of the association. The goodness of fit of the final model was checked by Hosmer–Lameshow test, at *p*-value of > 0.05. A two-sided *p*-value below 0.05 was considered statistically significant.

## Results

### Inclusion and general characteristics

Out of 30,506 individuals aged between 20 and 65 years old primarily registered in the KCHS through October 2016 to November 2018, 437 (1.43%) participants for whom a HGB measurements were not available, and 519 (1.70%) pregnant women were excluded. Data on 29,550 (96.87%) participants were included in the final analysis.

The mean ± SD age of the study population was 41.90 ± 11.88 years, and a female to male ratio of 1.75:1 was present. The distribution of subjects among different age groups was almost the same, with a slightly larger proportion in the middle age group of 35–49 years (38.77%). About three-quarters of participants were urban residents (73.54%), and others were from rural areas (26.46%). Arab ethnicity accounted for nearly half of the participants (48.65%), followed by Bakhtiari (22.06%), and Fars (18.68%) ethnicity. The majority of participants were married (82.71%). Regarding educational status, more than two-third of the study participants had primary (*n* = 8,899, 30.12%) or secondary (*n* = 11,445, 38.73%) levels of education, 5,441 (18.41%) were illiterate, and only 3,761 (12.73%) had university degrees and above. Only 8,731 (29.55%) subjects had an independent source of income, whether governmental, private, or self-employed, and 17,195 (58.19%) were housewives. In terms of wealth, individuals equally belonged to different quantiles from poorest to richest. Overall, more than two-third of individuals suffered from overweight (*n* = 11,064, 37.44%) or obesity (*n* = 8,814, 29.83%), and to some degree, 17,947 (60.73%) had an increased abdominal obesity. Currently or past, 3,253 (11.01%) and 1,582 (5.35%) of participants had ever smoked cigarettes and hookah in their lifetime, respectively, whereas, 641 (2.17%) had consumed alcohol, and 834 (2.82%) had used opium. Among all participants, the duration of night sleep was not enough or excessive in 12,994 (43.92%) subjects, and the amount of physical activity was classified as low, moderate, and high in 9,173 (31.04%), 12,597 (42.63%), and 7,703 (26.07%), respectively. Diabetes, hypertension, metabolic syndrome, and Chronic Kidney Disease (CKD) were present in 4,597 (15.56%), 5,883 (19.91%), 9,652 (32.66%), and 2,115 (7.2%) of the study population, respectively.

### Prevalence, severity, and types of anemia

**Fig. 1:**
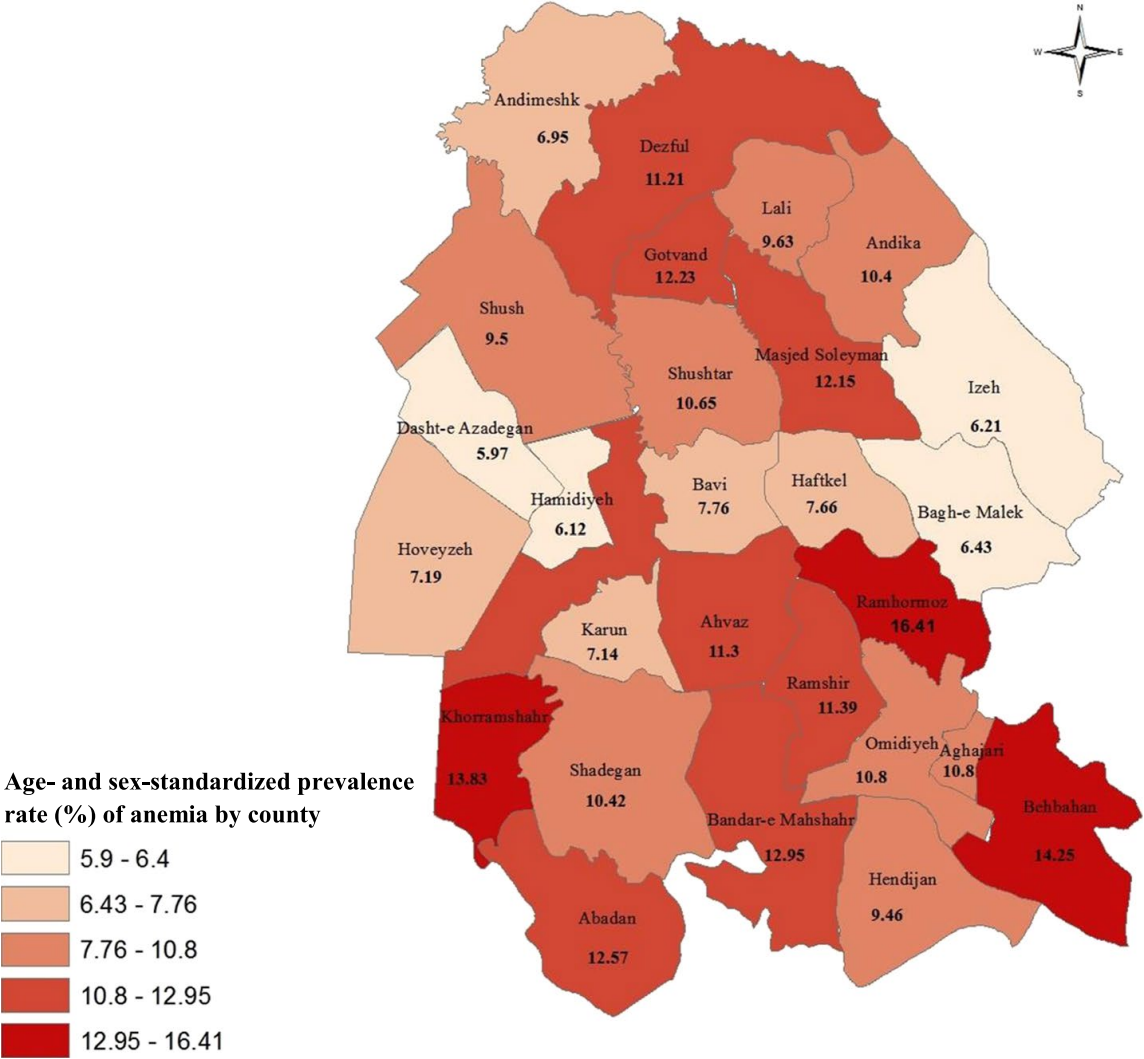
Visualizes the county-level age- and sex-standardized prevalence rate of anemia in the study population. There exists a wide regional variation of anemia level in Khuzestan, ranging from 5.97% in Dashte-e Azadegan to 16.41% in Ramhormoz

The mean ± SD HGB concentration of the study population was 13.75 ± 1.65 g/dL, and as expected, it was significantly lower in women than in men (12.94 ± 1.24 versus 15.15 ± 1.32, *p*-value < 0.0001). Using WHO cut-off values, 25,651 (86.8%) individuals were non-anemic (24,323 [82.3%] with normal HGB levels and 1,328 [4.5%] with polyglobulinemia) and 3,899 participants were found to be anemic. Thus, the overall and age- and sex-standardized prevalence rates of anemia were 13.19% (95% CI: 12.81–13.59%) and 10.86% (95% CI: 10.51–11.23%), respectively (Fig. 1).

Khuzestan map showing the county-level age- and sex-standardized prevalence rate of anemia in the study population, from 2016 to 2019.

**Fig. 2:**
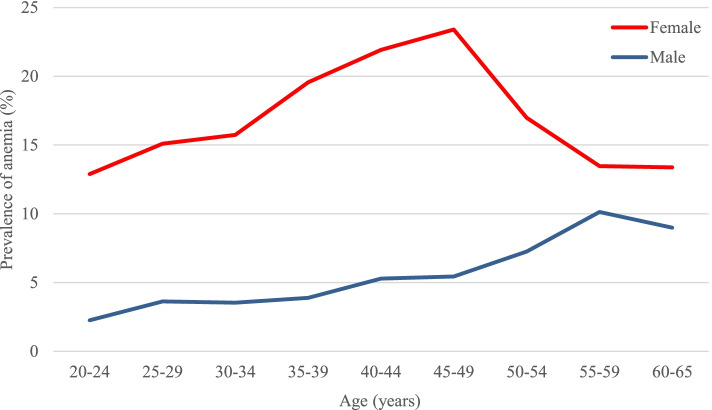
Line graphs illustrating the prevalence of anemia among females and males by age, Khuzestan, Iran, from 2016 to 2019

As shown in Fig. 2, important gender differences were observed in the prevalence of anemia. The anemia prevalence was notably higher among women compared to men in all age groups (adjusted: 17.08% versus 4.87%, *p*-value < 0.0001). However, the lines converge in older ages, as the prevalence rises in males and falls in females after middle-ages. In women, the anemia prevalence peaked in the 45–49 years, whereas the youngest subjects of 20–24-year group displayed comparably low rates (23.39% versus 12.88%, *p*-value < 0.0001). On the other hand, men experienced a steadily increasing trend in anemia prevalence with increasing age.

Mild degree anemia accounted for most of the cases with an age- and sex-standardized prevalence rate of 7.71% (95% CI: 7.40–8.03%), followed by moderate anemia in 2.98% (95% CI: 2.80–3.17%). Severe anemia was rare, with only 73 (0.17%, 95% CI: 0.14–0.22%) cases. Table 1 shows the age-specific proportion of anemia severity by gender. Of the anemic female population, a higher proportion suffered from moderate and severe anemia compared to the anemic men (34.82% versus 10.31%). The proportion of moderate and severe anemia was higher in the age group of 35–49 years (38.15%) compared with other age groups. Almost the same proportion was observed in females of 35–49 years (38.15%), but there was no significant age-specific difference in anemia severity among men.

**Table 1:** The severity of anemia in age-specific subgroups by gender among anemic population (*n* = 3,899) in Khuzestan, Iran, from 2016 to 2019

**Variables**	**Anemia severity **^**a**^			*P*-value ^*^
	**Mild**	**Moderate**	**Severe**	
**Overall**	**2,691 (69.02%)**	**1,135 (29.11%)**	**73 (1.87%)**	
**Total**				
Age groups (years)				< 0.0001
20–34	710 (70.44%)	288 (28.57%)	10 (0.99%)	
35–49	1,170 (64.82%)	585 (32.41%)	50 (2.77%)	
50–65	809 (74.98%)	257 (23.82%)	13 (1.2%)	
Gender				< 0.0001
Female	2,143 (65.18%)	1,075 (32.69%)	70 (2.13%)	
Male	548 (89.69%)	60 (9.82%)	3 (0.49%)	
**Female**				
20–34	615 (68.41%)	274 (30.48%)	10 (1.11%)	< 0.0001
35–49	997 (61.85%)	566 (35.11%)	49 (3.04%)	
50–65	529 (68.43%)	233 (30.14%)	11 (1.42%)	
**Male**				
20–34	95 (87.16%)	14 (12.84%)	0	0.544
35–49	173 (89.64%)	19 (9.84%)	1 (0.52%)	
50–65	280 (91.5%)	24 (7.84%)	2 (0.65%)	

Notes: *The chi-squared test was performed for comparison between the groups. Significance level was at *p*-value < 0.05

^a^, mild anemia: hemoglobin 11–11.9 g/dL in women and 11–12.9 g/dL in men; moderate anemia: hemoglobin 8–10.9 g/dL; severe anemia: hemoglobin < 8 g/dL

The types of anemia in age-specific subgroups by gender among the anemic population are presented in Table 2. According to the classification of anemias regarding the MCV and MCHC values, of those considered anemic, the highest proportion was related to normochromic/microcytic (50.65%), followed by hypochromic/microcytic (30.29%) and normochromic/normocytic (16.36%) anemia.

**Table 2:** The types of anemia in age-specific subgroups by gender based on the mean corpuscular volume and mean corpuscular hemoglobin concentration among anemic population (*n* = 3,899) in Khuzestan, Iran, from 2016 to 2019

**Variables**	**Anemia type**				*P*-value ^*^
	**Normochromic Normocytic**	**Normochromic Microcytic**	**Hypochromic Microcytic**	**Others **^**a**^	
**Overall**	**638 (16.36%)**	**1,975 (50.65%)**	**1,181 (30.29%)**	**105 (2.69%)**	
**Total**					
Age groups (years)					< 0.0001
20–34	155 (15.38%)	530 (52.58%)	296 (29.37%)	27 (2.68%)	
35–49	255 (14.13%)	962 (53.3%)	552 (30.58%)	36 (1.99%)	
50–65	227 (21.04%)	481 (44.58%)	331 (30.68%)	40 (3.71%)	
Gender					< 0.0001
Female	526 (16.00%)	1,724 (52.43%)	956 (29.08%)	82 (2.49%)	
Male	112 (18.33%)	251 (41.08%)	225 (36.82%)	23 (3.76%)	
**Female**					
20–34	145 (16.13%)	492 (54.73%)	238 (26.47%)	24 (2.67%)	0.001
35–49	227 (14.08%)	874 (54.22%)	477 (29.59%)	34 (2.11%)	
50–65	153 (19.79%)	357 (46.18%)	240 (31.05%)	23 (2.98%)	
**Male**					
20–34	10 (9.17%)	38 (34.86%)	58 (53.21%)	3 (2.75%)	< 0.0001
35–49	28 (14.51%)	88 (45.6%)	75 (38.86%)	2 (1.04%)	
50–65	74 (24.18%)	124 (40.52%)	91 (29.74%)	17 (5.56%)	

Notes: *The chi-squared test was performed for comparison between the groups. Significance level was at *p*-value < 0.05

^a^, Other types include: hypochromic/normocytic: 25 (0.64%); hyperchromic/microcytic: 49 (1.26%); hyperchromic/normocytic: 28 (0.72%); and hyperchromic macrocytic: 3 (0.08%)

### Factors associated with anemia

The crude and age- and sex-standardized prevalence rates of anemia by baseline characteristics are shown in detail in Table 3. The univariate analysis demonstrated that 17 variables were significantly associated with the age- and sex-standardized anemia status. Accordingly, anemia was significantly more prevalent among female sex, individuals aged 35–49 years, urban residents, Fars ethnicity, divorced or widowed category, illiterate educational levels, housewives, lower wealth status, underweight subjects, excessive night sleepers, those with moderate physical activity, and participants with CKD. Moreover, cigarettes or hookah smokers, opium users, alcohol drinkers, and participants with metabolic syndrome were negatively associated with anemia. There were no significant differences in relation to the other variables of the study.

**Table 3:** The crude and age- and sex-standardized prevalence rates of anemia by baseline characteristics (*n* = 3,899) in Khuzestan, Iran, from 2016 to 2019

**Variables**	**Frequency (n)**	**Anemia prevalence % (95% CI)**		*P*-value^*^
		**Crude**	**Age- and sex- standardized**	
**Overall**	**3,899 / 29,550**	**13.19 (12.81–13.59)**	**10.86 (10.51–11.23)**	
**Demographics**				
Gender ^**^				< 0.0001
Female	3,288 / 18,789	17.50 (16.96–18.05)	17.08 (16.52–17.66)	
Male	611 / 10,761	5.68 (5.26–6.13)	4.87 (4.47–5.30)	
Age categories (years) ^a ***^				< 0.0001
20–34	1,008 / 9,329	10.81 (10.19–11.45)	9.19 (8.64–9.76)	
35–49	1,805 / 11,456	15.76 (15.10–16.43)	13.43 (12.84–14.04)	
50–65	1,079 / 8,711	12.39 (11.71–13.10)	11.53 (10.87–12.23)	
Area of residence				< 0.0001
Rural	900 / 7,819	11.51 (10.82–12.24)	9.19 (8.58–9.85)	
Urban	2,999 / 21,731	13.80 (13.35–14.27)	11.54 (11.12–11.98)	
Ethnicity ^a^				0.028
Arab	1,902 / 14,377	13.23 (12.69–13.79)	10.66 (10.16–11.17)	
Bakhtiari	844 / 6,520	12.94 (12.15–13.78)	10.75 (10.01–11.54)	
Fars	766 / 5,521	13.87 (12.99–14.81)	11.98 (11.11–12.90)	
Others	380 / 3,078	12.35 (11.23–13.56)	10.12 (9.10–11.24)	
Marital status				< 0.0001
Single	404 / 3,601	11.22 (10.23–12.29)	8.54 (7.71–9.45)	
Married	3,257 / 24,441	13.33 (12.91–13.76)	11.20 (10.81–11.61)	
Divorced / Widowed	238 / 1,508	15.78 (14.03–17.71)	15.41 (13.59–17.43)	
**Socioeconomics variables**				
Educational Level ^b^				< 0.0001
Illiterate	805 / 5,441	14.80 (13.88–15.76)	13.50 (12.60–14.46)	
Primary	1,269 / 8,899	14.26 (13.55–15.00)	12.22 (11.55–12.92)	
Secondary	1,413 / 11,445	12.35 (11.76–12.96)	10.02 (9.48–10.58)	
Tertiary	411 / 3,761	10.93 (9.97–11.97)	8.88 (8.02–9.82)	
Occupational status ^c^				< 0.0001
Working	530 / 8,731	6.07 (5.59–6.59)	5.08 (4.63–5.56)	
Housewife	3,018 / 17,195	17.55 (16.99–18.13)	17.10 (16.52–17.71)	
Unemployed / Retired / Student	315 / 3,426	9.19 (8.27–10.21)	7.68 (6.83–8.64)	
Wealth status quartiles ^d^				0.001
Lowest	1,010 / 7,350	13.74 (12.97–14.55)	11.58 (10.85–12.35)	
Up to median	988 / 7,257	13.61 (12.84–14.42)	11.65 (10.91–12.44)	
Above median	1,014 / 7,843	12.93 (12.20–13.69)	10.25 (9.61–10.93)	
Highest	865 / 6,925	12.49 (11.73–13.29)	10.06 (9.37–10.79)	
**Individual factors**				
BMI categories ^e^				< 0.0001
Underweight	139 / 811	17.14 (14.70–19.89)	13.84 (11.65–16.37)	
Normal weight	1,146 / 8,724	13.14 (12.44–13.86)	10.71 (10.08–11.38)	
Overweight	1,319 / 11,064	11.92 (11.33–12.54)	9.71 (9.17–10.28)	
Obese	1,271 / 8,814	14.42 (13.70–15.17)	12.23 (11.56–12.94)	
Abdominal obesity ^f^				0.226
Normal	1,545 / 11,463	13.48 (12.87–14.12)	10.62 (10.07–11.19)	
Increased	2,332 / 17,947	12.99 (12.51–13.49)	11.06 (10.60–11.53)	
Ever cigarettes smoker ^g^				< 0.0001
Yes	223 / 3,253	6.86 (6.04–7.78)	5.40 (4.68–6.22)	
No	3,672 / 26,241	13.99 (13.58–14.42)	11.75 (11.36–12.15)	
Ever hookah smoker ^g^				< 0.0001
Yes	91 / 1,582	5.75 (4.71–7.01)	3.80 (3.03–4.76)	
No	3,804 / 27,912	13.63 (13.23–14.04)	11.51 (11.13–11.89)	
Ever opium user ^g^				< 0.0001
Yes	60 / 834	7.19 (5.63–9.16)	5.58 (4.28–7.24)	
No	3,835 / 28,660	13.38 (12.99–13.78)	11.07 (10.71–11.45)	
Ever alcohol drinker ^g^				< 0.0001
Yes	22 / 641	3.43 (2.27–5.16)	2.35 (1.52–3.64)	
No	3,873 / 28,853	13.42 (13.03–13.82)	11.20 (10.83–11.57)	
Sleeping duration ^h^				< 0.0001
Enough	2,174 / 16,522	13.16 (12.65–13.68)	10.85 (10.38–11.34)	
Not enough	1,325 / 10,551	12.56 (11.94–13.20)	10.14 (9.58–10.74)	
Excessive	397 / 2,443	16.25 (14.84–17.77)	13.79 (12.46–15.24)	
Physical activity tertiles ^i^				< 0.0001
Low	1,080 / 9,173	11.77 (11.13–12.45)	9.58 (8.99–10.21)	
Moderate	1,779 / 12,597	14.12 (13.53–14.74)	12.08 (11.51–12.68)	
High	1,032 / 7,703	13.40 (12.65–14.18)	10.64 (9.98–11.35)	
**Medical characteristics**				
Diabetes				0.128
Yes	616 / 4,597	13.40 (12.45–14.42)	11.64 (10.74–12.61)	
No	3,283 / 24,953	13.16 (12.74–13.58)	10.76 (10.38–11.16)	
Hypertension				0.193
Yes	789 / 5,883	13.41 (12.56–14.31)	11.42 (10.62–12.27)	
No	3,110 / 23,667	13.14 (12.72–13.58)	10.76 (10.37–11.17)	
Metabolic syndrome				< 0.0001
Yes	1165 / 9,652	12.07 (11.44–12.74)	9.83 (9.25–10.43)	
No	2,734 / 19,898	13.74 (13.27–14.23)	11.26 (10.83–11.72)	
CKD ^j^				< 0.0001
Yes	270 / 2,115	16.41 (14.89–18.05)	14.51 (13.06–16.10)	
Non-CKD / Unknown	3,622 / 27,376	12.95 (12.56–13.35)	10.66 (10.30–11.04)	

Abbreviations: *CI* confidence interval, *BMI* body mass index, *CKD* chronic kidney disease

Notes: *The chi-squared test was performed for comparison between the groups. Significance level was at *p*-value < 0.05

^**^Age-standardized

^***^Sex-standardized

Missing values, count (%): a, 54 (0.18); b, 4 (0.01); c, 198 (0.67); d, 175 (0.59); e, 137 (0.46); f, 140 (0.47); g, 56 (0.19); h, 34 (0.12); i, 77 (0.26); j, 59 (0.20ege)

A multiple logistic regression was performed to ascertain whether the explanatory variables found in univariate analyses could be further confirmed. As shown in Table 4, eleven associations for anemia were highlighted in the multivariable analysis: 1. gender, 2. age groups, 3. Body Mass Index (BMI) category, 4. occupation, 5. area of residence, 6. hookah smoking, 7. presence of CKD, 8. presence of metabolic syndrome, 9. night sleep duration, 10. marital status, and 11. ethnicity. The Nagelkerke pseudo-R2 for the model was 0.074 and the *p*-value for Hosmer–Lemeshow test was 0.107. In this analysis, gender, age groups, BMI category, occupation, and area of residence, were able to explain 7.0% of the variability in anemia status compared to 7.4% in the saturated model.

**Table 4:** Associated factors of the anemia prevalence in multivariable analysis in Khuzestan, Iran, from 2016 to 2019

**Variables**	**Anemia prevalence ratio **^**a**^		*P*-value^*^
	**Adjusted OR **^**b**^	**95% CI**	
**Demographics**			
Gender			
Female	3.17	2.68–3.76	< 0.0001
Male	Reference		
Age categories (years)			
20–34	Reference		
35–49	1.66	1.52–1.82	< 0.0001
50–65	1.27	1.14–1.42	< 0.0001
Area of residence			
Rural	Reference		
Urban	1.18	1.09–1.29	< 0.0001
Ethnicity			
Arab	0.95	0.87–1.05	0.334
Bakhtiari	0.91	0.81–1.01	0.081
Fars	Reference		
Others	0.83	0.72–0.95	0.008
Marital status			
Single	Reference		
Married	1.16	1.02–1.32	0.021
Divorced / Widowed	1.02	0.84–1.24	0.844
**Socioeconomics variables**			
Occupational status			
Working	Reference		
Housewife	1.26	1.07–1.48	0.006
Unemployed / Retired / Student	1.55	1.33–1.81	< 0.0001
**Individual factors**			
BMI categories			
Underweight	1.58	1.29–1.93	< 0.0001
Normal weight	Reference		
Overweight	0.80	0.73–0.87	< 0.0001
Obese	0.84	0.76–0.93	0.001
Ever hookah smoker			
Yes	0.67	0.53–0.83	< 0.0001
No	Reference		
Sleeping duration			
Enough	Reference		
Not enough	1.04	0.96–1.12	0.338
Excessive	1.19	1.06–1.35	0.004
**Medical characteristics**			
Metabolic syndrome			
Yes	0.87	0.80–0.95	0.002
No	Reference		
CKD			
Yes	1.27	(1.12–1.44)	< 0.0001
Non-CKD / Unknown	Reference		

Abbreviations: *CI* confidence interval, *OR* odds ration, *BMI* body mass index

Notes: ^a^, the ORs and 95% CIs were generated from the logistic regression analysis. Significance level was at p-value < 0.05

^b^, adjusted for gender, age groups, BMI categories, occupation, area of residence, hookah smoking, presence of CKD, presence of metabolic syndrome, night sleep duration, marital status, and ethnicity. (Nagelkerke pseudo-R^2^ = 0.074 and Hosmer–Lemeshow *p*-value = 0.107)

In the final model of logistic regression, the odds of anemia were more than threefold (OR: 3.17, 95% CI: 2.68–3.76) increased with women than men. Age was found to be significantly related to anemia with odds ratio of 1.66 (95% CI: 1.52–1.82) in the age group 35–49 years, and 1.27 (95% CI: 1.14–1.42) in the age group 50–65 years as compared with age group 20–34 years. Compared to normal BMI, individuals who were underweight were 1.58 times (95% CI: 1.29–1.93) as likely to have anemia. Regarding occupation, being unemployed or retired (OR: 1.55, 95% CI: 1.33–1.81), followed by being housewife (OR: 1.26, 95% CI: 1.07–1.48) was associated with higher prevalence of anemia than those who were working. And finally, there was a tendency for higher anemia prevalence in subjects living in urban areas than in rural areas (OR: 1.18, 95% CI: 1.09–1.29). Those suffering from CKD (OR: 1.27, 95% CI: 1.12–1.44), excessive night sleepers versus normal sleepers (OR: 1.19, 95% CI: 1.06–1.35), and married participants versus singles (OR: 1.16, 95% CI: 1.02–1.32) had only slightly increased likelihood of anemia. The observed negatively associated factors for anemia were hookah smoking (OR: 0.67, 95% CI: 0.53–0.83), metabolic syndrome (OR: 0.87, 95% CI: 0.80–0.95), overweight and obesity (OR: 0.80, 95% CI: 0.73–0.87, and OR: 0.84, 95% CI: 0.76–0.93, respectively), and belonging to minority ethnic groups (OR: 0.83, 95% CI: 0.72–0.95%). There was no difference between other remaining variables.

## Discussion

Anemia is a major global public health problem affecting about one-quarter of the world’s population [4]. The available data from the previous epidemiological studies indicate that the anemia status varies among populations depending on various factors. Therefore, we aimed at addressing the provincial and county-level prevalence of anemia, its severity and types in a large population-based study representative of the general adult population in Khuzestan province, as well as thoroughly investigating its association with the background characteristics of subjects.

### Anemia status (prevalence, severity, and subtypes) and associated factors

The prevalence of anemia among men and non-pregnant women aged between 20–65 years in Khuzestan was 10.86%, which is categorized of mild public health significance as per WHO classification and it is similar to high-income countries [1]. The most prevalent degree of anemia was mild (7.71%), followed by moderate (2.98%), and only 0.17% of all subjects were severely anemic. Our findings are in line with the results of the second national integrated micronutrient survey in Iran, which estimated that the anemia prevalence is 10.3% (9.6–11.0%) among adults 45–60 years [18]. In another a population-based cross-sectional study of 1,675 individuals aged 1–90 years in Mashhad, the prevalence of anemia was calculated to be 9.7% [19, 20].

Normochromic/microcytic followed by hypochromic/microcytic and normochromic/normocytic anemias in our study could point towards the fact that malnutrition, iron deficiency, thalassemia and chronic diseases are relevant causes for anemia in Khuzestan population [21]. About four fifth of anemic subjects had microcytosis in both women and men, but among them, men had higher proportion of hypochromia compared to women (36.82% versus 29.08%). The proportion of normochromic/normocytic anemia increased with age in both males and females, which is mostly related to anemia of chronic disease [21]. Similarly, these types of anemia were also reported as the most prevalent types of anemia in various studies [22–24].

Either as a risk or consequence of anemia, the parameters of female gender, age group of 35–49 years, belonging to the underweight category of BMI, being unemployed or retired, and living in urban areas were major determinants of anemia prevalence in our study according to multivariable analysis. These results are mostly in agreement with findings obtained from previous investigations. The prevalence of anemia was clearly higher among women totally (OR: 3.17). This is consistent with numerous studies, [18–20, 23, 25] and may be simply explained by biological vulnerability of the inevitable iron loss during menstruation and pregnancy in the reproductive age as well as dietary inadequacy. Regarding age, it is important to note that there were differences between the genders. Among men, there was a steady increase in the prevalence of anemia with advancing age. On the other hand, women particularly had a higher prevalence of anemia in age group of 35–49 years, and then experienced a downward trend. Aging process, inflammation, menopause, chronic diseases, and reduction in testosterone production, all may explain these observed differences. Next factor is nutrition and economic status which work synergically toward anemia. As such, we observed that low BMIs and undernourished individuals have an inverse relationship with anemia. Although low BMI may not necessarily imply poorer micronutrient intake, they are more likely to have other concomitant comorbidities and hence, poorer nutrition. Unlike most earlier studies, [8, 26] the two variables of lower educational levels and wealth index quantiles did not significantly influence the anemia prevalence in our model, however, the differences were meaningful in univariate analysis. This may be explained by the improved economic status of the region, and thereby, improved nutrition conditions, lower infection rates, better health-related access, and other favorable living conditions. Housewives were the most prevalent form of occupation among anemic population. The gender-specific variation of the sample composition could have affected this outcome. After adjustment, the respective odds were strongly lowered, but the effect of being unemployed or retired remained pretty much the same on the prevalence of anemia. These observations are repeated in a cross-sectional study among apparently healthy residents in Ethiopia [26]. Unlike most other studies, [26–28] inhabitants of urban regions in our study tended to be more affected by anemia. Similar associations with geographic location have been found in Maldives and Timor-Leste [28].

In addition to factors described above, our study demonstrated a minor but significant positive association between anemia status and suffering from CKD, night sleep duration, and marital status, as well as a negative association between anemia and factors including hookah smoking and presence of metabolic syndrome. As expected, participants with CKD were more likely to suffer from anemia, as anemia is a well-known complication of CKD, mainly due to absolute or relative erythropoietin deficiency [29]. Recently, few epidemiological studies have observed an association between either a long or a short sleep duration and anemia prevalence, [30–32] i.e. anemia is less prevalent in individuals who sleep between 7 and 9 h per night. In the present study, we found also that a long sleep duration independently predicts an increased prevalence of anemia. However, it is not possible to exactly determine the causality based on this cross-sectional data and further comprehensive longitudinal studies are needed. Furthermore, this association may be more likely because of other unmeasured confounding factors like poor sleep quality. To date, no definite underlying mechanism exists in this regard. Inflammation is one of the proposed biological pathways. It has been observed that the level of inflammatory markers including sensitivity C-reactive protein is higher in those with extreme sleep duration and also in anemic individuals [30, 31, 33]. Also, both disturbed sleep duration and anemia can cause serious health issues, like stroke, coronary heart disease and mortality events [34]. Therefore, it would be beneficial if our physicians are aware of this association and consider sleep problems in anemia treatment as a modifiable risk factor. In this study, the odds of having anemia in married individuals, after being adjusted, remained slightly higher when compared with their counterparts. However, Rwanda and south and southeast Asian countries demonstrated higher prevalence of anemia in widowed or divorced women of reproductive age, but still not the never married women [28, 35]. It could probably be hypothesized that women are susceptible to more menstruation, pregnancy and birth-related complications, resulting in recurrent blood loss, and men are predisposed to economic deprivation, poverty, and malnutrition less time spent on health services, as they are the main breadwinners of their family [36]. It is also important for policy makers to mention that the majority of women marry during adolescence for traditional reasons in Khuzestan, and therefore, are exposed to its complications earlier [37].

Hookah is a classical style of tobacco smoking, originating from the middle east. Here we also observed that hookah smokers were less likely to have anemia. Hookah has already been recognized as a stimulant of HGB production due to induced hypoxemia, [38] and the lower prevalence of anemia among hookah smokers in our study may be attributed to this reason. This result is also consistent with a study conducted in Taif city in Saudi Arabia, which was found that shisha or cigarette smoking cause increased HGB levels and secondary polycythemia [39]. Although the cigarette smoking contributed to the same correlation in univariate analysis, it was not found significant in multivariate model. We should know that in a shisha session (which usually takes 20–80 min), people inhale the same amount of over 100 cigarettes smoke and potentially exposes the smoker to more smoke over time [40]. Given a somehow similar mechanism to overweight or obesity, the presence of metabolic syndrome in a subject is another negative predictor of anemia prevalence. Other studies have also reported alterations in hematological parameters with a trend towards higher HGB concentrations in subjects with metabolic syndrome [41, 42]. And finally regarding ethnicity, we have to point out that there was no significant difference between Fars and Arabs or Bakhtiari in terms of anemia prevalence, who consist about 90% of our study population. However, a minor negative association was observed between anemia and being from minor ethnic groups (Lur, Turk, and Kurd). This may be of interest since Arabs are somehow genetically different from other Iranian ethnic groups compared to each other and Khuzestan province houses the largest population of Iranian Arabs [43].

### Strengths and limitations

The strength of this report comes from the use of a large population-based multi-ethnic sample size covering 29,550 adult men and non-pregnant women from all counties of the Khuzestan province, which makes it very likely reflective of the current anemia status, with precise point estimates, in the general adult population of the region. The reliability and validity of the results were assured by the standardized quality control measures in data collection, management, and analysis. As a result, we could successfully propose some recommendations according to the identified associated factors of anemia for policy makers to implement measures to improve pertinent health status.

However, several limitations should be taken into account when discussing these results. Firstly, the KCHS data were collected in a one-time period with no follow-up to assess the impact of any interventions. Owing to the cross-sectional nature of the KCHS, no time precedence was recognized between most of the associated factors and anemia, thereby, the temporal causality pathway remained unclear. Secondly, the results of this report are not generalizable to younger or older age groups than our included age category of 20–65 years. Thirdly, the response rate in women was higher in comparison to men, However, this was adjusted by reporting gender-specific prevalence rates in data analysis. Fourthly, the majority parts of the data collection process in KCHS was questionnaire-based and relied on the respondents’ memory, which unavoidably might have caused recall bias. Also, we acknowledge the presence of a few missing data, although they seem negligible. Fifth, despite several existing definitions and cut-off values of anemia, we utilized the one from WHO based on the HGB concentration alone. Finally, our study lacks certain important influential factors, as well as etiological factors, for further interpretation of the attributed risk of anemia. These include dietary patterns, iron indices, or vitamin levels, presence of infections, inherited HGB variants, underlying chronic diseases, and bleeding history. Thus, regarding the subtypes and probable causes for anemia, we could only speculate possibilities based on MCV and MCHC.

## Conclusions

The results presented here provide for the first time an overview of anemia prevalence and its characterization in the men and non-pregnant women aged between 20–65 years in Khuzestan province, Iran. Taken together, the overall prevalence of anemia in this study population was of mild public health significance according to WHO classification and similar to other high-income countries. Furthermore, more than two-third of these cases had mild anemia. The high proportion of normochromic/microcytic, hypochromic/microcytic, and normochromic/normocytic anemias in our study may point towards the fact that the main causes of anemia are anemias of iron deficiency, chronic disease, and thalassemia. A comparably higher rates of anemia were observed amongst women, individuals aged 35–49 years, underweights, unemployed or retired subjects, and urban resident. To a minor degree, being 50–65 years of age, housewife, excessive night sleeper, CKD sufferer, and married were also related to anemia. Our epidemiological findings could provide reliable evidence for effective intervention strategies to prevent and control anemia by targeting the observed vulnerable population, particularly through paying adequate clinical attention in terms of iron status and underlying pathological conditions.

## Supplementary Information

**Additional file 1.**

**Additional file 2.**

## Data Availability

The datasets used and analyzed during the current study are available from the corresponding author on reasonable request.

## Abbreviations

KCHS: Khuzestan comprehensive health study
HGB: Hemoglobin
WHO: World health organization
RBC: Red blood cell
GBD 2013: Global burden of diseases, injuries and risk factors 2013
YLD: Years lived with disability
NIMAD: National institute for medical research development
IBTO: Iranian blood transfer organization
DDRI: Digestive diseases research institute
TUMS: Tehran university of medical sciences
MCHC: Mean corpuscular hemoglobin concentration
MCV: Mean corpuscular volume
OR: Odds ratio
CKD: Chronic kidney disease
BMI: Body mass index
ELSA: English longitudinal study of ageing
