# Supplementary material for: Anemia prevalence, severity, types, and correlates among adult women and men in a multiethnic Iranian population: the Khuzestan Comprehensive Health Study (KCHS)
Source: BMC Public Health. 2022 Jan 25;22:168. doi: 10.1186/s12889-022-12512-6 (PMC8787906; doi:10.1186/s12889-022-12512-6)
Supplement: Supplementary file 1 — Additional file 1. [file 12889_2022_12512_MOESM1_ESM.docx]

| Additional file 1. The variables included in the report of anemia status from the KCHS database, Khuzestan, Iran, from 2016 to 2019. | | | |
| --- | --- | --- | --- |
| **Data category** | | **Variables** | **Categories** |
| **Questionnaire** |  |  |  |
|  | Demographics ^a^ |  |  |
|  |  | Age (years) | 20-34, 35-49, 50-65 |
|  |  | Gender | female, male |
|  |  | County |  |
|  |  | Area of residence | rural, urban |
|  |  | Ethnicity | Arab, Bakhtiari, Fars, Kurd, Lur, Turk |
|  |  | Marital status | married, single, divorced or widowed |
|  | Socioeconomics ^a^ |  |  |
|  |  | Education (years) | illiterate: 0, primary: 1-6, secondary: 7-12, tertiary: ≥13 |
|  |  | Occupation | housewife, working, unemployed or retired or student |
|  |  | Wealth score ^b^ | lowest, up to median, above median, highest |
|  | Medical history |  |  |
|  |  | Diabetes ^c^ |  |
|  |  | Hypertension ^d^ |  |
|  |  | Metabolic syndrome ^e^ |  |
|  |  | CKD ^f^ |  |
|  | Personal habits |  |  |
|  |  | Ever cigarettes smoker |  |
|  |  | Ever hookah smoker |  |
|  |  | Ever opium user |  |
|  |  | Ever alcohol drinker |  |
|  |  | Sleeping duration (hours) | not enough: <7, enough: 7-9, excessive: >9 |
|  |  | Physical activity ^g^ | low, moderate, high |
| **Anthropometric Measurements** | |  |  |
|  | | BMI (kg/m^2^) | underweight: <18.5, normal weight: 18.5-24.9, overweight: 25-29.9, obese: ≥30 |
|  |  | Abdominal obesity | normal, increased: WHR >0.85 in women and >0.90 in men |
| **Laboratory Tests** | |  |  |
|  | | HGB (g/dL) | Polyglobulinemia: >16 in women and >16.5 in men, Normal: 12-16 in women and 13-16.5 in men, mild anemia: 11-11.9 in women and 11-12.9 in men, moderate anemia: 8-10.9, severe anemia: <8 |
|  |  | MCV (fL) | microcytosis: <80, normocytosis: 80-100, and macrocytosis: >100 |
|  |  | MCHC (%) | hypochromia: <32.5, normochromia: 32.5-35.2, and hyperchromia: >35.2 |
|  |  | RDW (%) | normal <15, anisocytosis ≥15 |
|  | | Creatinine (mg/dL) |  |
|  | | | |
| **Abbreviations:** KCHS, Khuzestan comprehensive health study; CKD, chronic kidney disease; ; eGFR, Estimated glomerular filtration rate BMI, body mass index; WHR, waist to hip circumference ratio; HGB, hemoglobin; MCV, mean corpuscular volume; MCHC, mean corpuscular hemoglobin concentrations; RDW, red cell distribution width. | | | |
| **Definitions:** a, demographic and socioeconomic information were determined by self-identification.  b, wealth index was calculated by means of principal component analysis (PCA) on the household's possession of durables, access to basic services, and characteristics of the house in which living, and then it was categorized to quantiles from poorest to richest.([1](#_ENREF_1))  c, Diabetes was defined as a fasting plasma glucose ≥126 mg/dL, or consumption of glucose lowering medications, or a self-report of history of a physician-related diabetes diagnosis.  d, Hypertension was defined as a systolic blood pressure >140 mmHg or diastolic blood pressure >90 mmHg, or taking blood pressure lowering drugs, or a self-reported physician-related diagnosis of hypertension.  e. Metabolic syndrome was defined by the presence of any three of the Adult Treatment Panel III (ATP III) criteria,([2](#_ENREF_2)) as follows:  1. abdominal obesity (waist circumstance ≥88 cm in women and ≥102 cm in men),  2. serum triglycerides ≥150 mg/dL), or drug treatment for hypertriglyceridemia,  3. serum high density lipoprotein (HDL) cholesterol <50 mg/dL in women and <40 mg/dL in men, or using medications for low HDL cholesterol,  4. blood pressure ≥130/85 mmHg, or using antihypertensive drugs,  5. fasting plasma glucose (FPG) ≥100 mg/dL, or using antihyperglycemic drugs.  f. Since no data regarding albuminuria and radiological evaluation was available for accurate classification of subjects into CKD stages,([3](#_ENREF_3)) CKD was defined by an eGFR of less than 60 mL/min/1.73 m^2^, or being on dialysis, or reporting a past medical history of kidney transplantation. Each subject’s eGFR was calculated using the 4-variable Modification of Diet in Renal Disease (MDRD) equation:([4](#_ENREF_4))  MDRD equation: 186 × (Serum Creatinine)^-1.154^ × (age)^-0.203^ × 0.742 (if female)  g. The intensity of physical activities was calculated by the Metabolic Equivalent of Task (MET) score, defined as the ratio of one’s activity metabolic rate relative to their resting metabolic rate,([5](#_ENREF_5)) and it was expressed in tertiles from lowest to highest. | | | |

1. Smits J, Steendijk R. The international wealth index (IWI). Social Indicators Research. 2015;122(1):65-85.

2. Alberti K, Eckel RH, Grundy SM, Zimmet PZ, Cleeman JI, Donato KA, et al. Harmonizing the metabolic syndrome: a joint interim statement of the international diabetes federation task force on epidemiology and prevention; national heart, lung, and blood institute; American heart association; world heart federation; international atherosclerosis society; and international association for the study of obesity. Circulation. 2009;120(16):1640-5.

3. Levey AS, Coresh J, Balk E, Kausz AT, Levin A, Steffes MW, et al. National Kidney Foundation practice guidelines for chronic kidney disease: evaluation, classification, and stratification. Annals of internal medicine. 2003;139(2):137-47.

4. Manjunath G, Sarnak MJ, Levey AS. Prediction equations to estimate glomerular filtration rate: an update. Current opinion in nephrology and hypertension. 2001;10(6):785-92.

5. Haskell WL, Lee I-M, Pate RR, Powell KE, Blair SN, Franklin BA, et al. Physical activity and public health: updated recommendation for adults from the American College of Sports Medicine and the American Heart Association. Circulation. 2007;116(9):1081.

**Legend:**

**File name:** Additional file 1

**File format:** A table in .docx format

**Title of data:** The variables included in the report of anemia status from the KCHS database, Khuzestan, Iran, from 2016 to 2019.

**Description of data:** All required data for this report were extracted from the database of the KCHS and are outlined in Additional file 1 by the definitions and categories of each variable.
